# Diethyl 4-(biphenyl-4-yl)-2,6-dimethyl-1,4-di­hydro­pyridine-3,5-di­carboxyl­ate

**DOI:** 10.1107/S1600536814013294

**Published:** 2014-06-18

**Authors:** Scott A. Steiger, Anthony J. Monacelli, Chun Li, Janet L. Hunting, Nicholas R. Natale

**Affiliations:** a Department of Biomedical and Pharmaceutical Sciences, The University of Montana, 32 Campus Drive, Missoula, MT 59812, USA; b Department of Chemistry, Ithaca College, 953 Danby Road, Ithaca, NY 14850, USA

## Abstract

The title compound, C_25_H_27_NO_4_, has a flattened di­hydro­pyridine ring. The benzene and phenyl rings are synclinal to one another, forming a dihedral angle of 49.82 (8)°; the axis of the biphenyl rings makes an 81.05 (9)° angle to the plane of the di­hydro­pyridine ring. In the crystal, N—H⋯O hydrogen bonds link the mol­ecules into chain motifs running along the *a-*axis direction. The chains are cross-linked by C—H⋯O inter­actions, forming sheet motifs running slightly off the (110) plane, together with an intermolecular interaction between head-to tail biphenyl groups, thus making the whole crystal packing a three-dimensional network. Intra­molecular C—H⋯O hydrogen bonds are also observed.

## Related literature   

For general structure–activity relationship studies of 1,4-di­hydro­pyridines (DHPs) as calcium channel modulators, see: Bossert *et al.* (1981 ▶); Triggle (2003 ▶). For binding studies of DHPs to multiple drug resistant protein 1 (MDR1), see: Abe *et al.* (1995 ▶); Cole *et al.* (1989 ▶); Tasaki *et al.* (1995 ▶); Vanhoefer *et al.* (1999 ▶); Tolomero *et al.* (1994 ▶); Cindric *et al.* (2010 ▶).
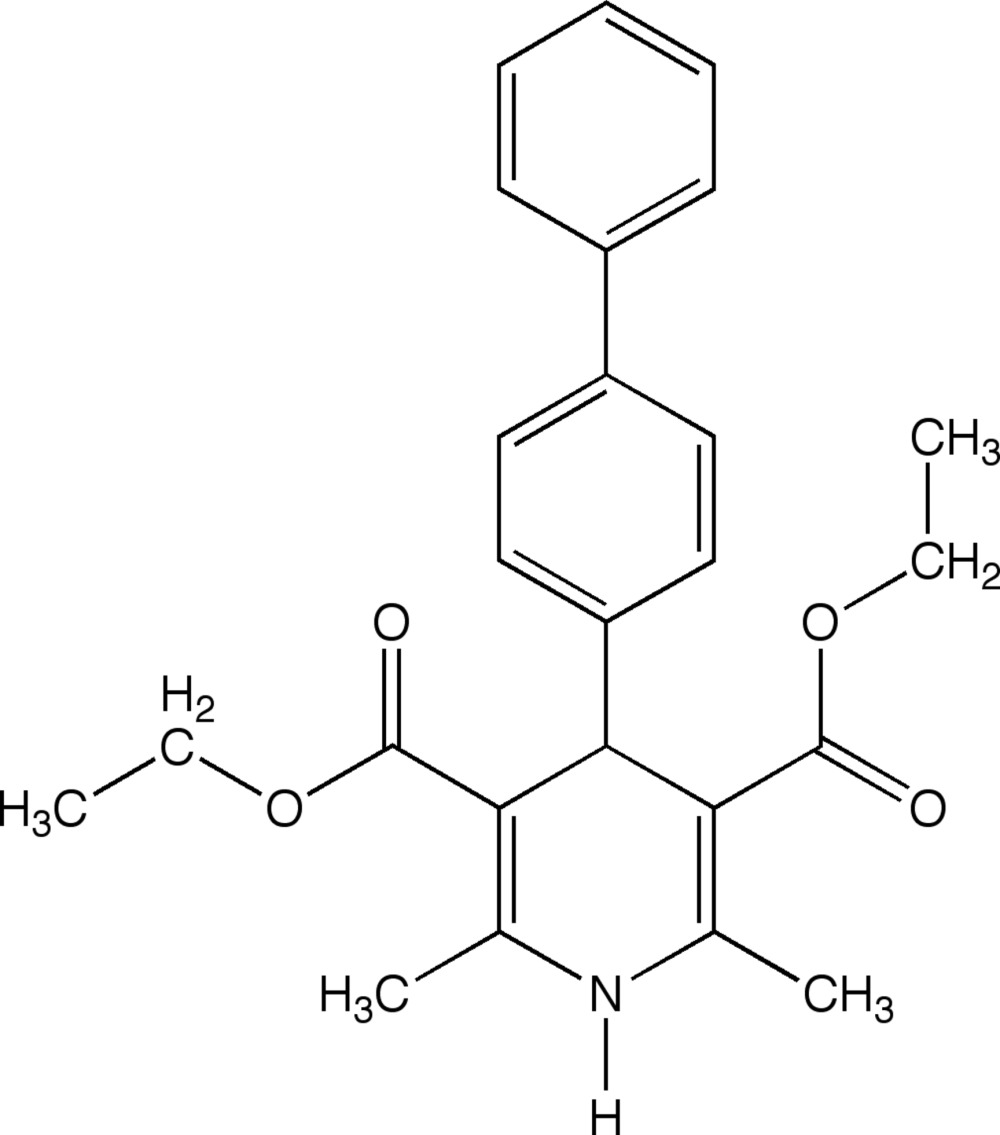


## Experimental   

### 

#### Crystal data  

C_25_H_27_NO_4_
*M* *_r_* = 405.47
Triclinic, 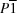

*a* = 7.3431 (3) Å
*b* = 10.6075 (4) Å
*c* = 13.8449 (6) Å
α = 85.762 (3)°
β = 88.124 (3)°
γ = 73.530 (2)°
*V* = 1031.25 (7) Å^3^
*Z* = 2
Mo *K*α radiation
μ = 0.09 mm^−1^
*T* = 100 K
0.15 × 0.14 × 0.13 mm

#### Data collection  

Bruker SMART BREEZE CCD diffractometer
Absorption correction: multi-scan (*SADABS*; Bruker, 2012 ▶) *T*_min_ = 0.919, *T*_max_ = 1.000
19956 measured reflections
4752 independent reflections
2983 reflections with *I* > 2σ(*I*)
*R*_int_ = 0.072

#### Refinement  

*R*[*F* ^2^ > 2σ(*F* ^2^)] = 0.062
*wR*(*F* ^2^) = 0.149
*S* = 1.02
4752 reflections
279 parameters
H atoms treated by a mixture of independent and constrained refinement
Δρ_max_ = 0.43 e Å^−3^
Δρ_min_ = −0.37 e Å^−3^

### 

Data collection: *APEX2* (Bruker, 2012 ▶); cell refinement: *SAINT* (Bruker, 2012 ▶); data reduction: *SAINT*; program(s) used to solve structure: *SHELXS97* (Sheldrick, 2008 ▶); program(s) used to refine structure: *SHELXL97* (Sheldrick, 2008 ▶); molecular graphics: *OLEX2* (Dolomanov *et al.*, 2009 ▶); software used to prepare material for publication: *OLEX2* (Dolomanov *et al.*, 2009 ▶).

## Supplementary Material

Crystal structure: contains datablock(s) I. DOI: 10.1107/S1600536814013294/zl2590sup1.cif

Structure factors: contains datablock(s) I. DOI: 10.1107/S1600536814013294/zl2590Isup2.hkl

Supporting information file. DOI: 10.1107/S1600536814013294/zl2590Isup3.cml

Additional supporting information:  crystallographic information; 3D view; checkCIF report

## Figures and Tables

**Table 1:** Hydrogen-bond geometry (Å, °)

*D*—H⋯*A*	*D*—H	H⋯*A*	*D*⋯*A*	*D*—H⋯*A*
C21—H21⋯O2^i^	0.95	2.50	3.256 (3)	137
C6—H6*A*⋯O3^ii^	0.98	2.59	3.452 (3)	147
C19—H19⋯O1	0.95	2.51	3.227 (3)	132
C13—H13*B*⋯O2	0.98	2.11	2.857 (3)	131
C8—H8*A*⋯O2^iii^	0.99	2.55	3.344 (3)	137
N1—H1⋯O3^ii^	0.91 (3)	2.03 (3)	2.938 (3)	173 (2)

Symmetry codes: (i) 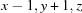; (ii) 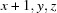; (iii) 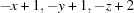.

**Fig. 1.**
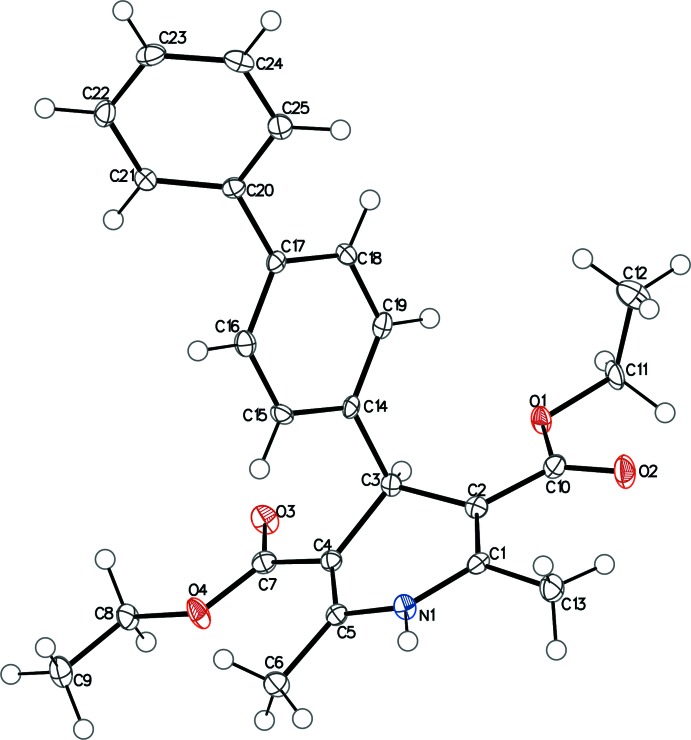
Crystal structure of the title compound with labeling and displacement ellipsoids drawn at the 50% probability level.

**Fig. 2.**
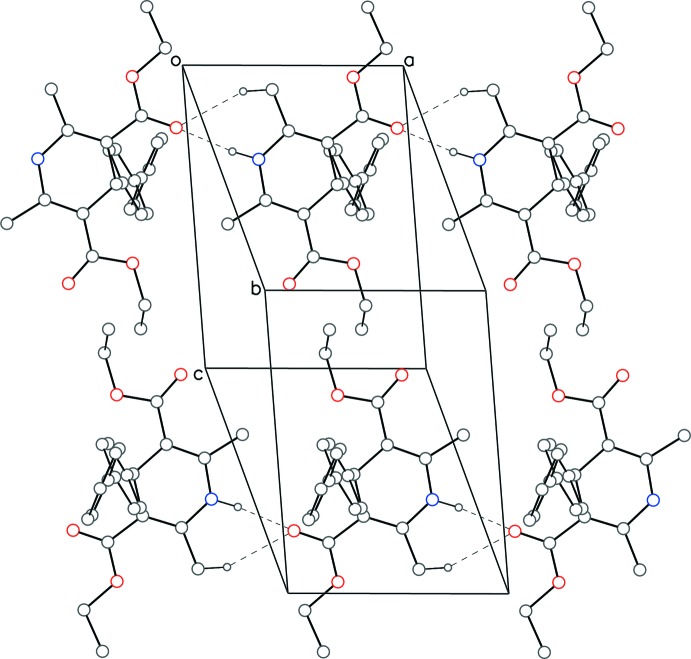
Packing diagram of the title compound, showing the intermolecular hydrogen bonds which form chain motifs running along the a axis. For the sake of clarity, H atoms not involved in H-bonds are removed.

**Fig. 3.**
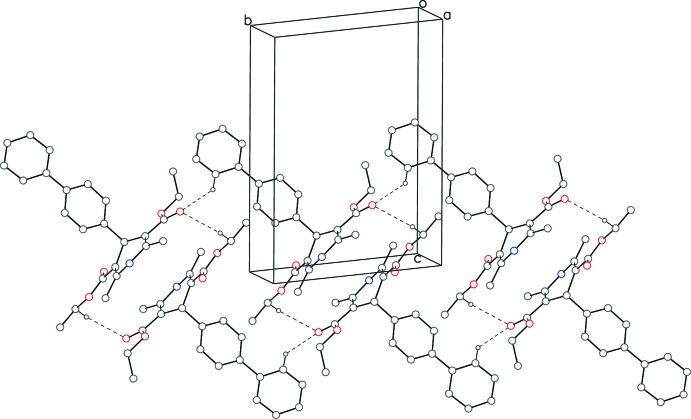
Packing diagram of the title compound, showing intermolecular C – H ··· O interactions in dashed lines which cross link the molecules into a sheet motif running slightly off the 110 plane. For the sake of clarity, H atoms not involved in the interactions are removed.

**Fig. 4.**
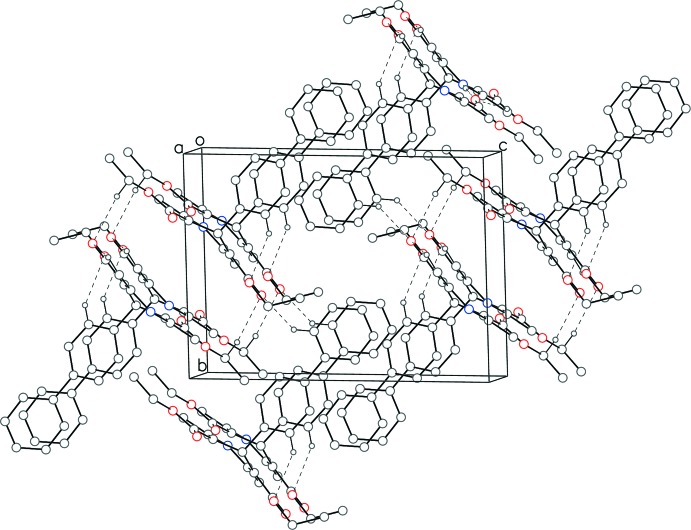
Packing diagram of the title compound. The intermolecular interactions form a three-dimensional network in the crystal packing. For the sake of clarity, H atoms not involved in the interactions are removed.

## supplementary crystallographic information

### S1. Comment

Hantzsch 1,4-di­hydro­pyridines (DHPs) are an extensively studied class of compounds that are known predominantly for their L-type voltage gated calcium channel modulation. (Bossert *et al.* 1981, Triggle 2003) There have been extensive structure-activity relationship (SAR) studies done on DHPs that have revealed the basic structural requirements for robust binding affinity to calcium channels. (Triggle 2003) Other studies in the field have shown that DHPs bind to multiple receptors, most notably the multiple drug resistant protein 1 (MDR1) (Abe *et al.* 1995, Cole *et al.* 1989, Tasaki *et al.* 1995, Vanhoefer *et al.* 1999, Tolomero *et al.* 1994, Cindric *et al.* 2010). Using established SAR more selective compounds can be designed for greater selectivity resulting in more clinically relevant compounds.

The title compound, C_25_H_27_NO_4_, has very similar structural features as other DHPs. Such features include a flattened boat conformation of the 1,4-DHP ring and two ester groups coplanar to the double bonds in the 1,4-DHP, with one carbonyl being *cis* and the other carbonyl being *trans* to the double bonds (Figure 1). Although the phenyl group attached at C(3) is still orthogonal to the bottom [C(1)—C(2)—C(4)—C(5)] of the 1,4-DHP ring [81.05 (9)°], it twists away from the N(1)—C(3) at an angle of 47.77 (8)°. The next phenyl ring twists again, with 49.82 (8)° from the center phenyl group, and becomes almost orthogonal to the N(1)—C(3) axis [12.97 (9)°]. Inter­molecular hydrogen bonds between N(1) – H(1) and O(3), together with the inter­molecular C(6) – H(6) ··· O(3) inter­actions, link the molecules into chain motifs running along the *a* axis (Figure 2). Two inter­molecular C – H ··· O inter­actions both from O(2) cross link the molecules into sheet motifs running slightly off the 110 plane (Figure 3). These inter­ations form a three-dimensional network in the cyrstal packing (Figure 4). There are two intra­molecular H-bonds observed in the molecule, C(19) – H(19) ··· O(1) and C(13) – H(13B) ··· O(2).

### S2.1. Synthesis and crystallization

An oven-dried 100 mL round bottom flask was charged with 1.90g of bi­phenyl-4-carbaldehyde, 2.86 g of ethyl aceto­acetate, 2.49 mL of 14.8M ammonium hydroxide, and a magenetic stir bar. The mixture was taken up in 50 mL of absolute ethanol, and the round bottom flask was fitted with a dean stark trap and heated to reflux while stirring. Reaction progress was monitored via TLC. Once the reaction was complete, excess solvent was removed via rotary evaporation. The solution was then purified via a silica column chromatography. The product was re-crystallized into white to yellow crystalline clumps with hexane and di­chloro­methane (yield = 1.24g , 3.06 mmol, 29.31%).

### S2.2. Refinement

Crystal data, data collection and structure refinement details are summarized in Table 1. The methyl H atoms were constrained to an ideal geometry, with C – H = 0.98 Å and *U*_iso_(H) = 1.5*U*_eq_(C), and were allowed to rotate freely about the C – C bonds. The rest of the H atoms were placed in calculated positions with C – H = 0.95 ~ 1.00 Å and refined as riding on their carrier atoms with *U*_iso_(H) = 1.2*U*_eq_(C). The positions of amine H atoms were determined from difference Fourier maps and refined freely along with their isotropic displacement parameters. One low-angle reflection was omitted from the refinement because its observed intensity was much lower than the calculated value as a result of being partially obscured by the beam stop.

### Crystal data

**Table:**

C_25_H_27_NO_4_	*Z* = 2
*M**_r_* = 405.47	*F*(000) = 432
Triclinic, *P*1	*D*_x_ = 1.306 Mg m^−^^3^
*a* = 7.3431 (3) Å	Mo *K*α radiation, λ = 0.71073 Å
*b* = 10.6075 (4) Å	Cell parameters from 5122 reflections
*c* = 13.8449 (6) Å	θ = 2.4–27.4°
α = 85.762 (3)°	µ = 0.09 mm^−^^1^
β = 88.124 (3)°	*T* = 100 K
γ = 73.530 (2)°	Prism, pale white
*V* = 1031.25 (7) Å^3^	0.15 × 0.14 × 0.13 mm

### Data collection

**Table:**

Bruker SMART BREEZE CCD diffractometer	2983 reflections with *I* > 2σ(*I*)
Radiation source: 2 kW sealed X-ray tube	*R*_int_ = 0.072
φ and ω scans	θ_max_ = 27.6°, θ_min_ = 2.0°
Absorption correction: multi-scan (*SADABS*; Bruker, 2012)	*h* = −9→9
*T*_min_ = 0.919, *T*_max_ = 1.000	*k* = −13→13
19956 measured reflections	*l* = −17→18
4752 independent reflections	

### Refinement

**Table:**

Refinement on *F*^2^	Primary atom site location: structure-invariant direct methods
Least-squares matrix: full	Hydrogen site location: mixed
*R*[*F*^2^ > 2σ(*F*^2^)] = 0.062	H atoms treated by a mixture of independent and constrained refinement
*wR*(*F*^2^) = 0.149	*w* = 1/[σ^2^(*F*_o_^2^) + (0.0537*P*)^2^ + 0.8331*P*] where *P* = (*F*_o_^2^ + 2*F*_c_^2^)/3
*S* = 1.02	(Δ/σ)_max_ = 0.001
4752 reflections	Δρ_max_ = 0.43 e Å^−^^3^
279 parameters	Δρ_min_ = −0.37 e Å^−^^3^
0 restraints	

### Special details

**Table:**

Geometry. All e.s.d.'s (except the e.s.d. in the dihedral angle between two l.s. planes) are estimated using the full covariance matrix. The cell e.s.d.'s are taken into account individually in the estimation of e.s.d.'s in distances, angles and torsion angles; correlations between e.s.d.'s in cell parameters are only used when they are defined by crystal symmetry. An approximate (isotropic) treatment of cell e.s.d.'s is used for estimating e.s.d.'s involving l.s. planes.
Refinement. Refinement of *F*^2^ against ALL reflections. The weighted *R*-factor *wR* and goodness of fit *S* are based on *F*^2^, conventional *R*-factors *R* are based on *F*, with *F* set to zero for negative *F*^2^. The threshold expression of *F*^2^ > σ(*F*^2^) is used only for calculating *R*-factors(gt) *etc*. and is not relevant to the choice of reflections for refinement. *R*-factors based on *F*^2^ are statistically about twice as large as those based on *F*, and *R*- factors based on ALL data will be even larger.

### Fractional atomic coordinates and isotropic or equivalent isotropic displacement parameters (Å^2^)

**Table:**

	*x*	*y*	*z*	*U*_iso_*/*U*_eq_	
O3	0.1263 (2)	0.73985 (17)	0.98682 (13)	0.0183 (4)	
O1	0.4574 (2)	0.44644 (15)	0.75695 (12)	0.0149 (4)	
O4	0.2751 (2)	0.84869 (17)	1.07429 (13)	0.0191 (4)	
N1	0.7805 (3)	0.68215 (19)	0.92579 (15)	0.0127 (5)	
O2	0.7739 (2)	0.38137 (18)	0.73785 (14)	0.0248 (5)	
C3	0.4394 (3)	0.6641 (2)	0.85636 (17)	0.0111 (5)	
H3	0.3408	0.6153	0.8619	0.013*	
C4	0.4521 (3)	0.7191 (2)	0.95378 (17)	0.0115 (5)	
C20	0.1965 (3)	1.0952 (2)	0.55708 (17)	0.0118 (5)	
C14	0.3787 (3)	0.7763 (2)	0.77713 (17)	0.0107 (5)	
C25	0.2602 (3)	1.0854 (2)	0.46091 (18)	0.0156 (5)	
H25	0.3384	1.0037	0.4406	0.019*	
C1	0.7918 (3)	0.5861 (2)	0.86166 (17)	0.0122 (5)	
C18	0.2529 (3)	0.8563 (2)	0.61659 (18)	0.0141 (5)	
H18	0.2085	0.8383	0.5569	0.017*	
C5	0.6197 (3)	0.7352 (2)	0.98050 (17)	0.0112 (5)	
C2	0.6293 (3)	0.5673 (2)	0.83079 (17)	0.0118 (5)	
C7	0.2713 (3)	0.7670 (2)	1.00548 (18)	0.0136 (5)	
C21	0.0758 (3)	1.2152 (2)	0.58379 (18)	0.0138 (5)	
H21	0.0290	1.2231	0.6485	0.017*	
C22	0.0236 (3)	1.3226 (2)	0.51739 (19)	0.0167 (6)	
H22	−0.0601	1.4031	0.5365	0.020*	
C17	0.2607 (3)	0.9835 (2)	0.63075 (17)	0.0107 (5)	
C16	0.3313 (3)	1.0043 (2)	0.71876 (18)	0.0144 (5)	
H16	0.3398	1.0897	0.7301	0.017*	
C6	0.6587 (3)	0.8067 (2)	1.06364 (18)	0.0163 (6)	
H6A	0.7962	0.7883	1.0711	0.025*	
H6B	0.6033	0.7768	1.1233	0.025*	
H6C	0.6021	0.9017	1.0508	0.025*	
C19	0.3093 (3)	0.7555 (2)	0.68872 (18)	0.0139 (5)	
H19	0.3004	0.6701	0.6776	0.017*	
C15	0.3893 (3)	0.9031 (2)	0.78989 (18)	0.0125 (5)	
H15	0.4374	0.9204	0.8488	0.015*	
C13	0.9930 (3)	0.5135 (3)	0.8374 (2)	0.0204 (6)	
H13A	1.0506	0.5717	0.7965	0.031*	
H13B	0.9946	0.4360	0.8025	0.031*	
H13C	1.0654	0.4853	0.8972	0.031*	
C8	0.0994 (3)	0.9041 (2)	1.12773 (18)	0.0152 (5)	
H8A	0.0608	0.8337	1.1667	0.018*	
H8B	−0.0041	0.9496	1.0825	0.018*	
C10	0.6336 (3)	0.4574 (2)	0.77147 (18)	0.0146 (5)	
C9	0.1404 (4)	1.0005 (2)	1.19262 (19)	0.0195 (6)	
H9A	0.1838	1.0672	1.1532	0.029*	
H9B	0.2395	0.9535	1.2386	0.029*	
H9C	0.0246	1.0433	1.2283	0.029*	
C23	0.0929 (4)	1.3132 (2)	0.42313 (19)	0.0177 (6)	
H23	0.0603	1.3878	0.3781	0.021*	
C24	0.2102 (3)	1.1940 (2)	0.39484 (19)	0.0168 (6)	
H24	0.2564	1.1868	0.3300	0.020*	
C11	0.4471 (4)	0.3420 (2)	0.69640 (18)	0.0176 (6)	
H11A	0.3249	0.3213	0.7091	0.021*	
H11B	0.5507	0.2616	0.7141	0.021*	
C12	0.4631 (4)	0.3793 (3)	0.59046 (19)	0.0245 (6)	
H12A	0.4454	0.3093	0.5526	0.037*	
H12B	0.5891	0.3912	0.5763	0.037*	
H12C	0.3654	0.4618	0.5733	0.037*	
H1	0.893 (4)	0.696 (3)	0.9411 (19)	0.023 (8)*	

### Atomic displacement parameters (Å^2^)

**Table:**

	*U*^11^	*U*^22^	*U*^33^	*U*^12^	*U*^13^	*U*^23^
O3	0.0103 (9)	0.0255 (10)	0.0221 (11)	−0.0085 (8)	0.0015 (7)	−0.0084 (8)
O1	0.0152 (9)	0.0113 (9)	0.0194 (10)	−0.0046 (7)	0.0007 (7)	−0.0057 (7)
O4	0.0115 (9)	0.0269 (10)	0.0210 (10)	−0.0064 (8)	0.0055 (7)	−0.0145 (8)
N1	0.0078 (10)	0.0152 (11)	0.0168 (12)	−0.0054 (9)	0.0013 (8)	−0.0049 (9)
O2	0.0154 (9)	0.0238 (10)	0.0328 (12)	0.0018 (8)	−0.0007 (8)	−0.0158 (9)
C3	0.0091 (11)	0.0108 (12)	0.0145 (13)	−0.0040 (9)	−0.0004 (10)	−0.0026 (10)
C4	0.0122 (12)	0.0108 (12)	0.0110 (13)	−0.0026 (9)	0.0003 (9)	0.0000 (9)
C20	0.0106 (12)	0.0127 (12)	0.0127 (13)	−0.0044 (10)	−0.0018 (10)	−0.0002 (10)
C14	0.0068 (11)	0.0106 (12)	0.0130 (13)	0.0005 (9)	0.0019 (9)	−0.0018 (10)
C25	0.0126 (12)	0.0170 (13)	0.0181 (14)	−0.0047 (10)	−0.0010 (10)	−0.0039 (11)
C1	0.0108 (12)	0.0119 (12)	0.0126 (13)	−0.0016 (10)	−0.0005 (10)	0.0021 (10)
C18	0.0143 (12)	0.0145 (13)	0.0128 (14)	−0.0018 (10)	−0.0022 (10)	−0.0052 (10)
C5	0.0126 (12)	0.0093 (11)	0.0118 (13)	−0.0038 (9)	−0.0001 (10)	0.0011 (10)
C2	0.0131 (12)	0.0111 (12)	0.0120 (13)	−0.0050 (10)	0.0003 (10)	0.0006 (10)
C7	0.0142 (12)	0.0131 (12)	0.0136 (14)	−0.0044 (10)	−0.0007 (10)	0.0012 (10)
C21	0.0149 (12)	0.0138 (12)	0.0126 (13)	−0.0033 (10)	−0.0010 (10)	−0.0028 (10)
C22	0.0154 (13)	0.0113 (12)	0.0229 (15)	−0.0020 (10)	−0.0035 (11)	−0.0040 (11)
C17	0.0076 (11)	0.0116 (12)	0.0119 (13)	−0.0013 (9)	0.0018 (9)	−0.0011 (10)
C16	0.0161 (13)	0.0114 (12)	0.0168 (14)	−0.0051 (10)	0.0004 (10)	−0.0032 (10)
C6	0.0125 (12)	0.0205 (13)	0.0167 (14)	−0.0050 (10)	−0.0001 (10)	−0.0040 (11)
C19	0.0129 (12)	0.0091 (12)	0.0191 (14)	−0.0017 (10)	0.0003 (10)	−0.0020 (10)
C15	0.0131 (12)	0.0147 (12)	0.0109 (13)	−0.0050 (10)	−0.0022 (10)	−0.0033 (10)
C13	0.0133 (13)	0.0238 (14)	0.0220 (15)	−0.0003 (11)	0.0000 (11)	−0.0078 (12)
C8	0.0102 (12)	0.0200 (13)	0.0145 (14)	−0.0024 (10)	0.0040 (10)	−0.0048 (11)
C10	0.0147 (13)	0.0121 (12)	0.0156 (14)	−0.0016 (10)	−0.0009 (10)	0.0002 (10)
C9	0.0163 (13)	0.0194 (14)	0.0230 (16)	−0.0041 (11)	0.0036 (11)	−0.0089 (11)
C23	0.0221 (14)	0.0155 (13)	0.0163 (14)	−0.0075 (11)	−0.0065 (11)	0.0048 (11)
C24	0.0172 (13)	0.0244 (14)	0.0108 (13)	−0.0091 (11)	−0.0004 (10)	−0.0011 (11)
C11	0.0228 (14)	0.0110 (12)	0.0203 (15)	−0.0052 (11)	0.0002 (11)	−0.0081 (11)
C12	0.0253 (15)	0.0329 (16)	0.0195 (15)	−0.0135 (13)	−0.0007 (12)	−0.0067 (12)

### Geometric parameters (Å, º)

**Table:**

O3—C7	1.219 (3)	C21—C22	1.382 (3)
O1—C10	1.354 (3)	C22—H22	0.9500
O1—C11	1.458 (3)	C22—C23	1.385 (4)
O4—C7	1.340 (3)	C17—C16	1.396 (3)
O4—C8	1.459 (3)	C16—H16	0.9500
N1—C1	1.383 (3)	C16—C15	1.385 (3)
N1—C5	1.383 (3)	C6—H6A	0.9800
N1—H1	0.91 (3)	C6—H6B	0.9800
O2—C10	1.217 (3)	C6—H6C	0.9800
C3—H3	1.0000	C19—H19	0.9500
C3—C4	1.525 (3)	C15—H15	0.9500
C3—C14	1.536 (3)	C13—H13A	0.9800
C3—C2	1.527 (3)	C13—H13B	0.9800
C4—C5	1.356 (3)	C13—H13C	0.9800
C4—C7	1.464 (3)	C8—H8A	0.9900
C20—C25	1.398 (3)	C8—H8B	0.9900
C20—C21	1.396 (3)	C8—C9	1.506 (3)
C20—C17	1.484 (3)	C9—H9A	0.9800
C14—C19	1.397 (3)	C9—H9B	0.9800
C14—C15	1.392 (3)	C9—H9C	0.9800
C25—H25	0.9500	C23—H23	0.9500
C25—C24	1.388 (4)	C23—C24	1.388 (3)
C1—C2	1.352 (3)	C24—H24	0.9500
C1—C13	1.500 (3)	C11—H11A	0.9900
C18—H18	0.9500	C11—H11B	0.9900
C18—C17	1.395 (3)	C11—C12	1.500 (4)
C18—C19	1.389 (3)	C12—H12A	0.9800
C5—C6	1.502 (3)	C12—H12B	0.9800
C2—C10	1.468 (3)	C12—H12C	0.9800
C21—H21	0.9500		
			
C10—O1—C11	115.95 (18)	C5—C6—H6B	109.5
C7—O4—C8	117.92 (18)	C5—C6—H6C	109.5
C1—N1—H1	115.9 (17)	H6A—C6—H6B	109.5
C5—N1—C1	123.2 (2)	H6A—C6—H6C	109.5
C5—N1—H1	119.4 (17)	H6B—C6—H6C	109.5
C4—C3—H3	108.3	C14—C19—H19	119.1
C4—C3—C14	110.48 (18)	C18—C19—C14	121.8 (2)
C4—C3—C2	110.09 (19)	C18—C19—H19	119.1
C14—C3—H3	108.3	C14—C15—H15	119.2
C2—C3—H3	108.3	C16—C15—C14	121.6 (2)
C2—C3—C14	111.20 (19)	C16—C15—H15	119.2
C5—C4—C3	119.3 (2)	C1—C13—H13A	109.5
C5—C4—C7	124.8 (2)	C1—C13—H13B	109.5
C7—C4—C3	115.4 (2)	C1—C13—H13C	109.5
C25—C20—C17	121.4 (2)	H13A—C13—H13B	109.5
C21—C20—C25	118.4 (2)	H13A—C13—H13C	109.5
C21—C20—C17	120.2 (2)	H13B—C13—H13C	109.5
C19—C14—C3	121.1 (2)	O4—C8—H8A	110.5
C15—C14—C3	121.9 (2)	O4—C8—H8B	110.5
C15—C14—C19	117.0 (2)	O4—C8—C9	106.34 (19)
C20—C25—H25	119.7	H8A—C8—H8B	108.7
C24—C25—C20	120.5 (2)	C9—C8—H8A	110.5
C24—C25—H25	119.7	C9—C8—H8B	110.5
N1—C1—C13	112.5 (2)	O1—C10—C2	112.0 (2)
C2—C1—N1	118.8 (2)	O2—C10—O1	121.3 (2)
C2—C1—C13	128.7 (2)	O2—C10—C2	126.7 (2)
C17—C18—H18	119.6	C8—C9—H9A	109.5
C19—C18—H18	119.6	C8—C9—H9B	109.5
C19—C18—C17	120.8 (2)	C8—C9—H9C	109.5
N1—C5—C6	112.8 (2)	H9A—C9—H9B	109.5
C4—C5—N1	118.6 (2)	H9A—C9—H9C	109.5
C4—C5—C6	128.6 (2)	H9B—C9—H9C	109.5
C1—C2—C3	119.2 (2)	C22—C23—H23	120.2
C1—C2—C10	120.9 (2)	C22—C23—C24	119.6 (2)
C10—C2—C3	119.9 (2)	C24—C23—H23	120.2
O3—C7—O4	121.7 (2)	C25—C24—C23	120.3 (2)
O3—C7—C4	124.1 (2)	C25—C24—H24	119.9
O4—C7—C4	114.2 (2)	C23—C24—H24	119.9
C20—C21—H21	119.5	O1—C11—H11A	109.1
C22—C21—C20	120.9 (2)	O1—C11—H11B	109.1
C22—C21—H21	119.5	O1—C11—C12	112.4 (2)
C21—C22—H22	119.9	H11A—C11—H11B	107.9
C21—C22—C23	120.2 (2)	C12—C11—H11A	109.1
C23—C22—H22	119.9	C12—C11—H11B	109.1
C18—C17—C20	122.8 (2)	C11—C12—H12A	109.5
C18—C17—C16	117.5 (2)	C11—C12—H12B	109.5
C16—C17—C20	119.7 (2)	C11—C12—H12C	109.5
C17—C16—H16	119.3	H12A—C12—H12B	109.5
C15—C16—C17	121.3 (2)	H12A—C12—H12C	109.5
C15—C16—H16	119.3	H12B—C12—H12C	109.5
C5—C6—H6A	109.5		
			
N1—C1—C2—C3	8.3 (3)	C14—C3—C2—C10	−87.0 (3)
N1—C1—C2—C10	−173.2 (2)	C25—C20—C21—C22	−1.3 (3)
C3—C4—C5—N1	−10.0 (3)	C25—C20—C17—C18	−50.9 (3)
C3—C4—C5—C6	169.1 (2)	C25—C20—C17—C16	129.5 (2)
C3—C4—C7—O3	16.6 (3)	C1—N1—C5—C4	−17.3 (3)
C3—C4—C7—O4	−161.2 (2)	C1—N1—C5—C6	163.4 (2)
C3—C14—C19—C18	179.7 (2)	C1—C2—C10—O1	173.1 (2)
C3—C14—C15—C16	−178.9 (2)	C1—C2—C10—O2	−6.4 (4)
C3—C2—C10—O1	−8.3 (3)	C18—C17—C16—C15	−0.8 (3)
C3—C2—C10—O2	172.2 (2)	C5—N1—C1—C2	18.3 (4)
C4—C3—C14—C19	−163.0 (2)	C5—N1—C1—C13	−160.6 (2)
C4—C3—C14—C15	16.7 (3)	C5—C4—C7—O3	−171.8 (2)
C4—C3—C2—C1	−31.2 (3)	C5—C4—C7—O4	10.5 (3)
C4—C3—C2—C10	150.2 (2)	C2—C3—C4—C5	32.1 (3)
C20—C25—C24—C23	−1.3 (4)	C2—C3—C4—C7	−155.7 (2)
C20—C21—C22—C23	−0.9 (4)	C2—C3—C14—C19	74.4 (3)
C20—C17—C16—C15	178.8 (2)	C2—C3—C14—C15	−105.9 (2)
C14—C3—C4—C5	−91.1 (3)	C7—O4—C8—C9	−174.9 (2)
C14—C3—C4—C7	81.1 (2)	C7—C4—C5—N1	178.6 (2)
C14—C3—C2—C1	91.6 (3)	C7—C4—C5—C6	−2.3 (4)

### Hydrogen-bond geometry (Å, º)

**Table:**

*D*—H···*A*	*D*—H	H···*A*	*D*···*A*	*D*—H···*A*
C21—H21···O2^i^	0.95	2.50	3.256 (3)	137
C6—H6*A*···O3^ii^	0.98	2.59	3.452 (3)	147
C19—H19···O1	0.95	2.51	3.227 (3)	132
C13—H13*B*···O2	0.98	2.11	2.857 (3)	131
C8—H8*A*···O2^iii^	0.99	2.55	3.344 (3)	137
N1—H1···O3^ii^	0.91 (3)	2.03 (3)	2.938 (3)	173 (2)

Symmetry codes: (i) *x*−1, *y*+1, *z*; (ii) *x*+1, *y*, *z*; (iii) −*x*+1, −*y*+1, −*z*+2.
